# Dissociation between Mature Phenotype and Impaired Transmigration in Dendritic Cells from Heparanase-Deficient Mice

**DOI:** 10.1371/journal.pone.0035602

**Published:** 2012-05-10

**Authors:** Sandrine Benhamron, Inna Reiner, Eyal Zcharia, Mizhir Atallah, Amir Grau, Israel Vlodavsky, Dror Mevorach

**Affiliations:** 1 The Laboratory for Cellular and Molecular Immunology, Department of Medicine, Rheumatology Research Center, Hadassah-Hebrew University Medical Center, Jerusalem, Israel; 2 Sharett Institute of Oncology, Hadassah-Hebrew University Medical Center, Ein Kerem, Jerusalem, Israel; 3 Cancer and Vascular Biology Research Center, Bruce Rappaport Faculty of Medicine, Technion, Haifa, Israel; University of Lyon, France

## Abstract

To reach the lymphatics, migrating dendritic cells (DCs) need to interact with the extracellular matrix (ECM). Heparanase, a mammalian endo-β-D-glucuronidase, specifically degrades heparan sulfate proteoglycans ubiquitously associated with the cell surface and ECM. The role of heparanase in the physiology of bone marrow-derived DCs was studied in mutant heparanase knock-out *(Hpse*-KO) mice. Immature DCs from Hpse-KO mice exhibited a more mature phenotype; however their transmigration was significantly delayed, but not completely abolished, most probably due to the observed upregulation of MMP-14 and CCR7. Despite their mature phenotype, uptake of beads was comparable and uptake of apoptotic cells was more efficient in DCs from *Hpse*-KO mice. Heparanase is an important enzyme for DC transmigration. Together with CCR7 and its ligands, and probably MMP-14, heparanase controls DC trafficking.

## Introduction

Migrating dendritic cells (DCs) in different stages of maturation, and after antigen uptake, enter the lymphatic circulation where secondary lymphoid tissue chemokines (SLC/CCL21) and MIP-3-β/CCL19, acting via upregulated CCR7, have crucial roles [1], [2], [3], [4]. However, to reach the lymphatics, migrating DCs need to interact with, degrade, and transmigrate the extracellular matrix (ECM). Upon transmigration, factors released from the degraded ECM [5] may interact with DCs and influence their stimulatory capacity.

The ECM provides an essential physical barrier between cells and tissues, as well as a scaffold for cell growth, migration, differentiation, and survival. It undergoes continuous remodeling during development and in a variety of pathological conditions, including wound healing, inflammation, and cancer [6]. Heparan sulfate side chains of heparan sulfate proteoglycans bind major components of the ECM as well as multitude growth factors, chemokines, and cytokines. Cleavage of heparan sulfate side chains is therefore expected, not only to alter the structural integrity of the ECM, but also to release and modulate the activities of heparan sulfate-bound biological mediators [7].

Heparanase is an endo-β-D-glucuronidase capable of cleaving heparan sulfate side chains at a limited number of sites [8], [9], [10]. Through degradation of heparan sulfate, heparanase has been shown to facilitate ECM invasion by metastatic cancer cells [11], [12], as well as passage of immune cells such as macrophages [13], lymphocytes [5], [14], [15], and DCs into their target tissues, as shown recently by us [16]. Heparanase is synthesized by monocytes and early immature DCs as a preformed latent protein. It remains active in immature- and mature DCs, and upon DC maturation it accumulates in membrane extensions, most likely to allow ECM degradation.

We generated heparanase-deficient (*Hpse*-KO) mice in order to further study the role of heparanase in dendritic and other cells in health and disease. The mice, which showed complete lack of heparanase gene expression and enzymatic activity, developed normally, as was recently shown [17]. The present study focuses on examination of the role of heparanase in DCs from *Hpse*-KO mice, with regard to phenotype, phagocytosis of apoptotic cells, migration, and transmigration.

## Results

To confirm complete disruption of the *Hpse* gene, expression of heparanase mRNA derived from different tissues of wild-type (WT) and *Hpse*-KO mice was examined by real-time PCR using specific primers. The primers were designed to amplify the 5′, middle, and 3′ regions of the heparanase gene. Heparanase mRNA and enzymatic activity were detected in samples derived from *wt* mice, but not in *Hpse*-KO mice [17].

### Heparanase is Expressed in Bone Marrow-derived Dendritic Cells from *Hpse*-WT but not *Hpse*-KO Mice

**Figure 1:**
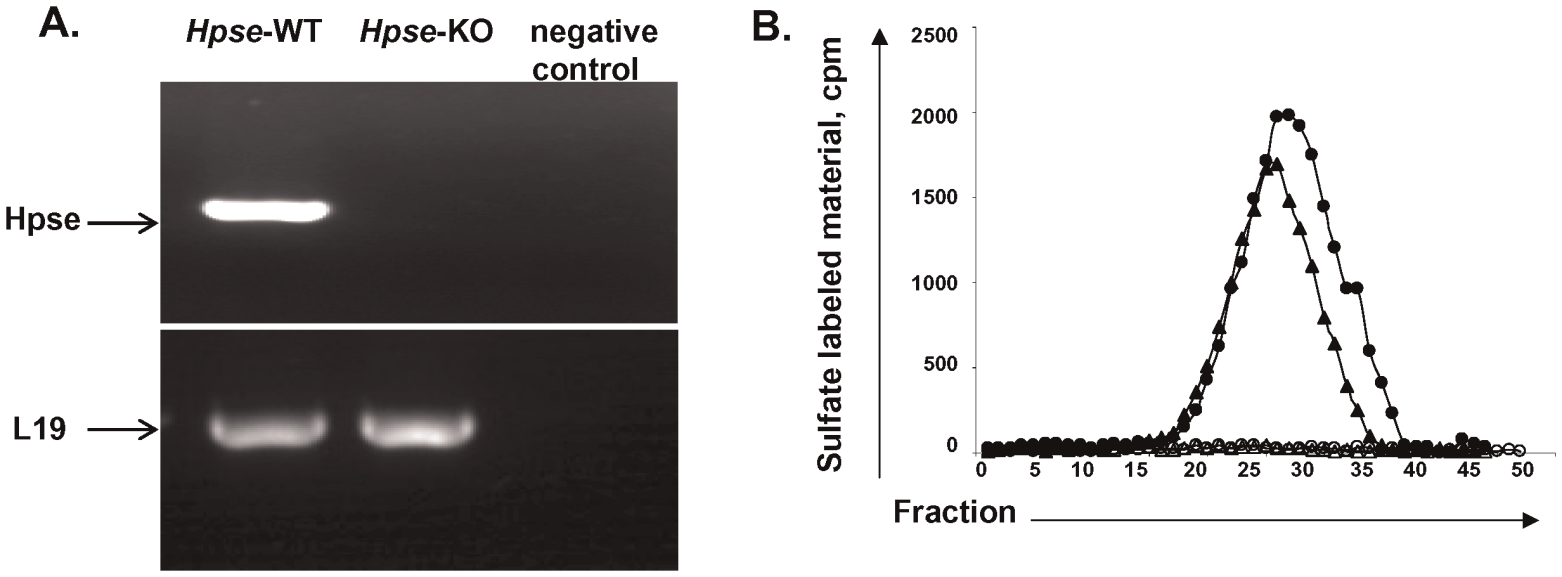
Heparanase is not expressed and not active in BMDCs from *Hpse*-KO mice. *A*. Heparanase RT-PCR. RNA was extracted from BMDCs of *Hpse-*WT and *Hpse*-KO mice. Primers specific for heparanase were used for RT-PCR (see Materials and Methods). Negative control represents RT-PCR without primer of heparanase. L-19 is shown for comparison. *B*. Heparanase Enzymatic Activity. Heparanase activity was determined in lysates of iBMDCs (closed circles) and mBMDCs (closed triangles) from *Hpse-*WT, and iBMDCs (opened circles) and mBMDCs (opened triangles) from *Hpse*-KO mice. Low-molecular-weight labeled degradation fragments eluted toward the V_t_ of the column (peak II, fractions 15–30, 0.5<K_av_<0.75) were previously shown to be fragments of heparan sulfate. In contrast, sulfate-labeled material released from ECM by proteolytic enzymes or nondegraded heparan sulfate is of much higher molecular weight, and eluted next to the void volume (peak I, fractions two-eight).The data shown are representative of three experiments.

RNA from *Hpse-*WT and *Hpse*-KO mice was extracted and heparanase gene expression was evaluated by RT-PCR. Figure 1A shows the presence of RNA in bone marrow-derived DCs (BMDCs) from *Hpse-*WT mice vs. no expression of heparanase RNA in BMDCs from *Hpse*-KO mice. We then sought to further verify that lack of heparanase is associated with lack of enzymatic activity [16]. Heparanase was active in immature BMDCs from *Hpse-*WT mice but there was no activity in immature BMDCs from *Hpse*-KO mice (Fig. 1B).

### Comparable Yield and Morphological Appearance of DCs from *Hpse*-WT and *Hpse*-KO Mice

Splenic dendritic cells had a comparable phenotype in both *Hpse*-KO and *Hpse*-WT (Table 1). As shown, there was a clear representation of all DC subpopulations. Mature DC number, lymphoid cell number, and lymphoid DC number were decreased in *Hpse*-KO mice, and plasmacytoid cell number was increased in *Hpse-*KO mice [18], however, these tendencies did not reach statistical significance.

**Figure 2:**
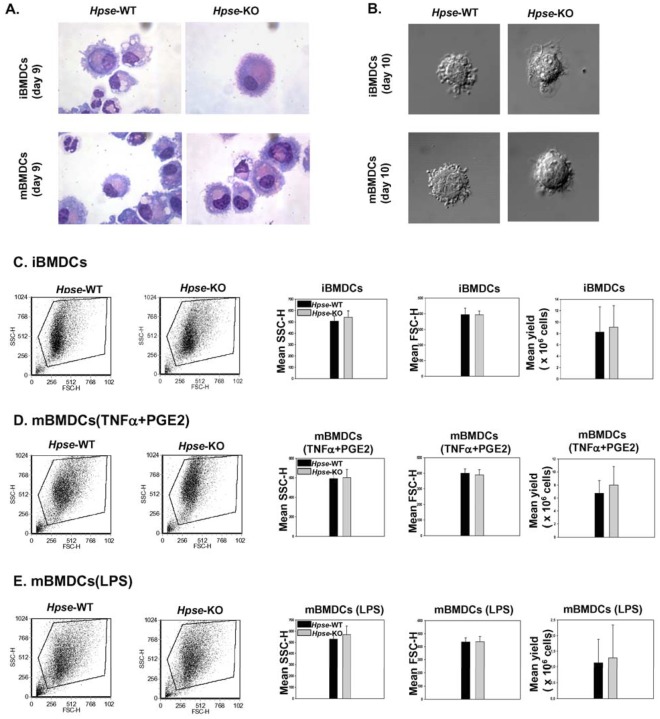
Morphologic parameters of iBMDCs and mBMDCs are comparable in *Hpse*-WT and *Hpse*-KO mice. *A*. Wright staining of BMDCs from *Hpse-*WT and *Hpse*-KO mice. *B* **.** Nomarsky view via confocal microscopy of BMDCs from *Hpse-*WT *and Hpse*-KO mice (zoom 60×5, Olympus IX70, Center Valley, PA). *C*. Representative dot plot analysis of side scatter (SSCH) and forward scatter (FSCH), and yield of iBMDCs. Average from five experiments. *D* and *E*. The same for mBMDCs, matured with mTNFα + PGE2 (*D*), or LPS (*E*).

We then compared the morphological appearance of BMDCs derived from *Hpse*-KO vs. *Hpse*-WT. No significant differences were observed using light and confocal microscopy (Figs. 2A and 2B). Flow cytometry analysis verified similar size and granularity (Figs. 2C, 2D, and 2E). Likewise, the yields of iBMDCs and mBMDCs from *Hpse*-WT and *Hpse*-KO mice were comparable (Figs. 2C, 2D, and 2E) despite a slight tendency to higher yield in the *Hpse*-KO.

**Table 1:** DC subpopulations in the spleen of wild type (*Hpse-*WT) and heparanase-deficient mice (*Hpse*-KO).

	*Hpse-*WT	*Hpse*-KO	*P values*
**Dendritic cells (CD11c^+^)**	3.64±0.33	3.18±0.09	0.27
**Mature DCs (CD11c^+^ IA/IE^+^)**	3.29±0.09	2.67±0.001	0.07
**Lymphoid DCs (CD11c^+^ CD8α^+^)**	1.05±0.24	0.76±0.14	0.31
**Myeloid DCs (CD11c^+^ CD11b^+^)**	1.90±0.14	1.54±0.03	0.16
**Plasmacytoid DCs (CD11c^+^ B220^+^)**	0.68±0.01	0.87±0.23	0.46

The average and standard deviation of four spleens is shown.

### Expression of Maturation and Migration Molecules is Higher in DCs from *Hpse*-KO Mice

**Figure 3:**
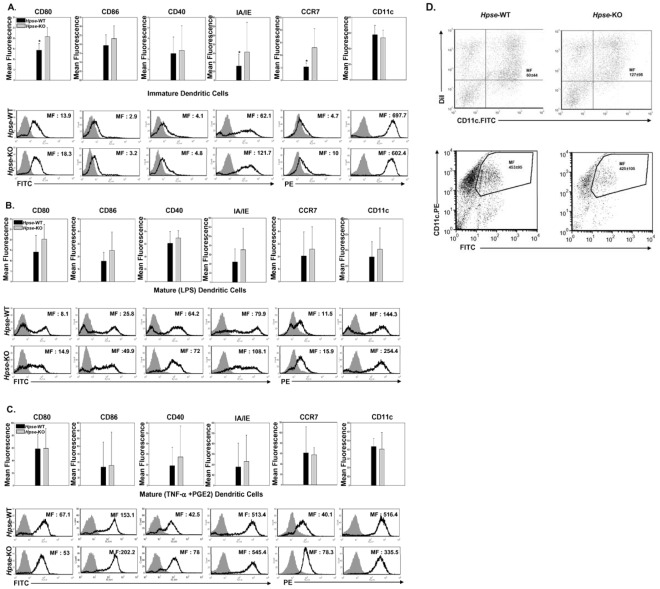
BMDCs from *Hpse*-KO mice have a phenotype with increased expression of maturation molecules and CCR7, and phagocytose apoptotic cells more efficiently. *A*, *B*, and *C*. *(A)* iBMDCs and (*B*) mBMDCs matured with LPS, and (*C*) mBMDCs matured with mTNFα + PGE2, from *Hpse-*WT and *Hpse*-KO mice, are stained for CD80, CD86, CD40, IA/IE, CCR7, and CD11c (black line), and for isotype control of each marker (gray-filled curves). Mean fluorescence (MF) is the average of five experiments. Asterisk indicates p<0.02, t-test. *D.* Uptake of apoptotic cells and FITC-labeled beads by iBMDC from *Hpse-*WT and *Hpse*-KO mice. *Upper panel.* Apoptotic cells were stained with DiI before the interaction with iBMDCs. 2 hours after the interaction with apoptotic cells, iBMDCs were stained with CD11c.FITC. Mean fluorescence (MF) of double positive cells is indicated. Phagocytosis was verified using confocal microscopy (not shown). *Lower panel.* As an additional control, FITC-labeled beads were offered to CD11c. PE-stained iBMDCs (MF) of double positive cells are indicated.

Dendritic cells were examined for expression of IA/IE and costimulatory molecules (Fig. 3). CD80 was significantly elevated in iBMDCs from *Hpse*-KO mice vs. *Hpse*-WT (Fig. 3A, 20.85±4.24 versus 14.28±3.29, p = 0.05, t-test), and there was a slight tendency toward increased CD86 and CD40 expression that did not reach statistical significance. IA/IE was also significantly increased in *Hpse*-KO mice (Fig. 3A, 63.15±66.39 versus 31.92±26.74, p = 0.03, t-test). In addition, CCR7 showed, similarly to CD80 and IA/IE, higher expression in *Hpse*-KO mice, although with borderline statistical significance mainly due to a greater standard deviation (Fig. 3A, 13±7.64 versus 5.37±1.53, p = 0.06, t-test). CD11c, as a control molecule less related to maturation and migration, was comparable in *Hpse*-KO and *Hpse*-WT mice (Fig. 3A). Upon exposure to LPS as a maturation factor, there was a tendency in most experiments for higher levels of CD80, IA/IE, and CCR7 (Fig. 3B), that reached statistical significance just when standardization was used taking *Hpse-*WT mice as 100% (20.29±7.18 versus 14.1±7.98, p = 0.06; 70.58±47.39 versus 44.35±28.15, p = 0.06; and 25.85±17.87 versus 20.61±19.09, p = 0.02, t-test, respectively). Using an additional maturation factor, a cocktail of 30 ng/ml TNFα and 1 µg/ml PGE2, CD80 and CD86 levels in *Hpse*- WT mice reached levels comparable to those in *Hpse*-KO mice. However, there was still a tendency in most experiments for higher CCR7 and IA/IE in DCs from *Hpse*-KO that did not reach statistical significant difference due to a large standard deviation (Fig. 3C, 231.85±250.6 versus 178.18±227.36, p = 0.08 for IA/IE, t-test). CD86 and CD40, in response to LPS as well as to 30 ng/ml mTNFα and 1 µg/ml PGE2, were less clearly increased (Figs. 3B and 3C).

Taken together, DCs in *Hpse*-KO mice showed a more mature phenotype, both in the so-called immature state, and in response to maturation signals. Interestingly, migration molecule CCR7 was highly expressed in both *Hpse*-KO iBMDCs and mBMDCs.

### Apoptotic Cells are More Efficiently Phagocytosed by iDCs from *Hpse*-KO Mice

We presumed that due to the mature phenotype seen in iDCs from Hpse-KO mice, phagocytosis of apoptotic cells will be decreased [19]. To our surprise, iDCs from Hpse-KO mice phagocytosed apoptotic cells more efficiently (p<0.0001). FITC-labeled beads were equally phagocytosed (Fig. 3D). Thus, despite a “mature” phenotype, iDCs from Hpse-KO mice were highly capable of apoptotic cell phagocytosis. Indeed, and not as generally appreciated, LPS-matured DCs were reported to maintain antigen capture via receptor-mediated endocytosis [20].

### Cytokine Secretion from iDCs and mDCs is Comparable in DCs from *Hpse*-WT and *Hpse*-KO Mice

**Figure 4:**
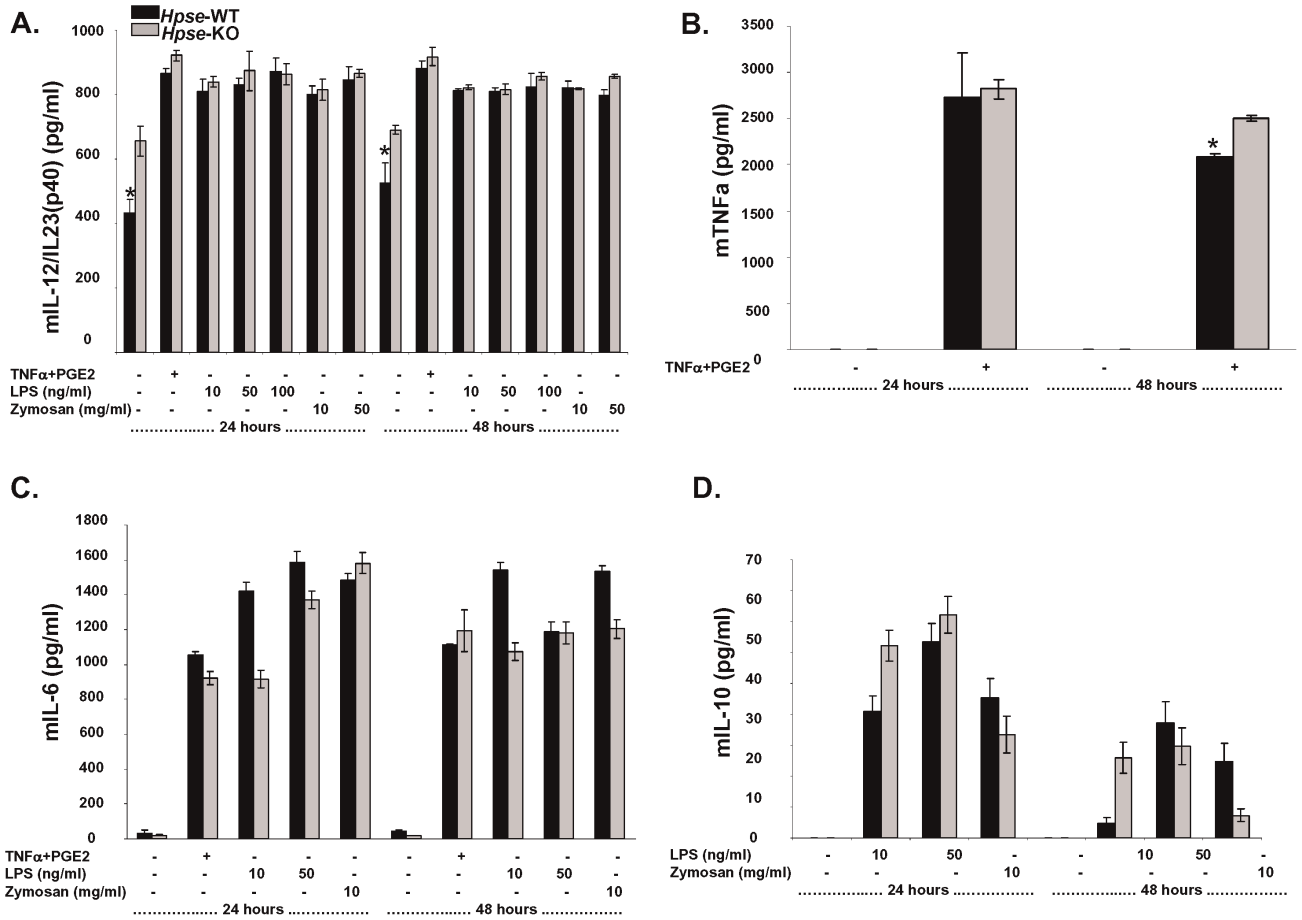
Cytokine secretion from iDCs and mDCs is comparable in DCs from *Hpse*-WT and *Hpse*-KO mice. *A*, *B*, *C* and *D*. Following DC maturation, supernatant culture was collected and analyzed by ELISA for (*A*) murine IL-12/IL-23(p40), (*B*) murine TNFα (*C*) murine IL-6, and (*D*) murine IL-10 production. Asterisk indicates p<0.02, t-test.

We were interested to know whether, despite their more mature phenotype, heparanase-deficient DCs are actually “semi-mature” DCs. Interestingly, E-cadherin-stimulated DCs failed to release immunostimulatory cytokines, but exhibited a mature phenotype with increased levels of MHC-class II and costimulatory molecules [21]. These “mature” DCs were suggested to be “semi-mature” tolerizing DCs, as the expected cytokine profile was observed. In contrast, as shown in Figure 4, DCs from *Hpse*-KO mice secreted cytokines in a comparable way to those from *Hpse*-WT mice, excluding the possibility of a “semi-mature” phenotype.

### Heparanase is Needed for *in vitro* Transmigration

**Figure 5:**
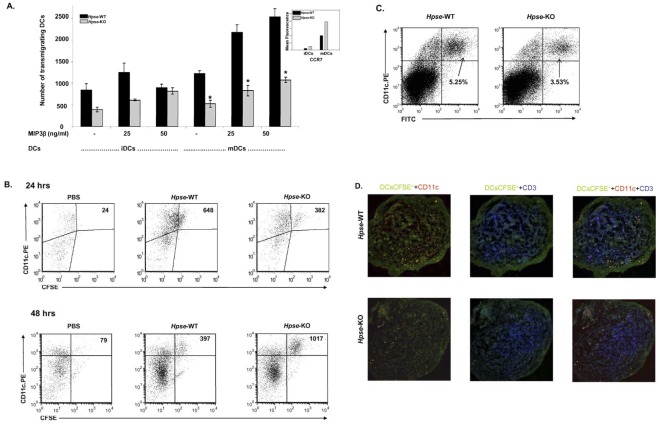
Heparanase is needed for in vitro and in vivo transmigration. *A*. *In vitro* transmigration of iBMDCs or mBMDCs from *Hpse-*WT (black bars) and *Hpse*-KO mice (gray bars). Cells were incubated in transwells coated with Matrigel (15 µg/50 µl DDW) 20–24 h at 37°C. Cells were incubated with the lower chamber medium containing RPMI, RPMI with 25 ng/ml MIP3β or RPMI with 50 ng/ml MIP3β. Representative of three experiments. Asterisk indicates p<0.02, t-test. *B. In vivo* DC transmigration. Flow cytometry. Dot plot of CD11c^+^ and CFSE^+^ cells from a popliteal lymph node following foot pad injection of PBS, or, 2×10^6^ mBMDCs from *Hpse-*WT, or 2×10^6^ mBMDCs *from Hpse*-KO mice. mBMDCs were stained with CFSE and injected SQ into the footpads of *Hpse-*WT mice. 24 hours or 48 hours after injection, the regional popliteal lymph node was extracted and analyzed for the presence of CFSE+ cells by flow cytometry. CyoCount beads were used to evaluate cells that had migrated to the popliteal lymph nodes. Representative of three experiments, (p<0.001). *C. In vivo* FITC skin painting. Dot plot of double positive CD11c-PE+ and FITC+ cells from inguinal lymph nodes. The shaved abdomen was painted with FITC solution. 24 hours after the skin painting, the inguinal lymph node was extracted and analyzed for the presence of CFSE+ cells by flow cytometry. *D.* Indirect immunofluorescence of frozen sections. Regional popliteal lymph node frozen section of the same experiment in *C*. Confocal microscopy of lymph nodes stained as described in Methods. CFSE-migrating DCs (green), CD11c migrating (yellow merged), local DCs (red), and T cells (blue), are seen.

Our previous study showed that heparanase plays an important role in the transmigration of mature monocyte-derived DCs [16]. We were interested to study the transmigration of iBMDCs and mBMDCs from *Hpse*-KO and *Hpse-*WT mice. We looked at BMDC transmigration in an ECM-coated two-chamber model. Figure 5A shows that iBMDC and mBMDC transmigration from *Hpse*-KO mice was significantly inhibited (p<0.001) compared to transmigration of iBMDCs and mBMDCs from *Hpse*-WT mice, both in the presence and absence of 25 ng/ml and 50 ng/ml MIP3β. Interestingly, despite a significantly lower transmigration rate, CCR7 expression was significantly increased (p<0.001) in both iBMDCs and mBMCs from *Hpse*-KO mice (Fig. 5A). Taken together, transmigration was significantly impaired but partially compensated, possibly due to upregulation of CCR7. We were then interested to see whether this transmigration defect is seen *in vivo*.

### Heparanase is Needed for *in vivo* DC Transmigration

2×10^6^ mBMDCs from *Hpse*-KO and *Hpse-*WT mice were stained with 2.5 µM CFSE and injected into the footpads of *Hpse-*WT mice. After 24 and 48 hours, popliteal lymph nodes (2 per mouse) were extracted, pooled, counted, and analysed by FACS for the presence of cells that were double-positive for CD11c-PE and CFSE.

CytoCount beads were used for evaluation of BMDC migration rates. As can be seen in a representative experiment (one of three) 24 hours following the injection, on the gated DCs, 648 events of CD11c+CFSE+ cells from *Hpse-*WT mice migrated to the popliteal lymph nodes, compared to only 382 events of CD11c+CFSE+ cells from *Hpse*-KO mice (Fig. 5B); thus a 28–52% reduction in migratory DCs was seen in *Hpse*-KO (p<0.0001). The total cell count in the popliteal lymph nodes from mice that had been injected with mBMDCs from *Hpse-*WT mice was 1.82×10^6^ cells, compared to the only slightly reduced total cell count in popliteal lymph nodes from *Hpse-*KO mice that were injected with PBS, which was 1.62×10^6^ cells. That suggests the possibility that immune response and cell expansion are only a slightly altered following DC migration. Interestingly, despite the reduced number of mBMDCs from *Hpse*-KO mice that reached the lymph nodes at 24 hours, almost the same number of cells, 1.6×10^6^, were counted in the nodes. To verify this observation, lymph nodes were sampled 24 hours following injection, and viewed using confocal microscopy. As shown in Figure 5D, fewer CFSE-labeled DCs arrived in the T cell zones of the regional popliteal lymph nodes.

On the other hand, 48 hours following the injection, on the gated DCs, 397 events of CD11c+CFSE+ cells from *Hpse-*WT mice were found in the popliteal lymph nodes, compared to 1017 events of CD11c+CFSE+ cells from *Hpse*-KO mice (Fig. 5B). In conclusion, whereas a delay in the arrival of injected CD11c+CFSE+ cells from *Hpse*-KO mice was seen at 24 hours, an accumulation and inhibition of natural decline in the number of CD11c+CFSE+ cells was seen 48 hours following the injection.

In order to further verify that DC migration was not influenced by the act of injecting the DCs into the footpads with possible mechanical disruption of the basement membrane, we used an additional transmigration assay in which a needle is not used: mouse abdominal fur was shaved, and FITC was painted onto the skin, as described elsewhere [22]. Tissue-resident DCs that pick up the dye and migrate to the superficial inguinal nodes were counted 24 hours later. As shown in Figure 5C, similarly to the foot pad transmigration assay, in *Hpse-*WT mice, 5.25% of lymph node cells were CD11c+ DCs carrying FITC whereas only 3.53% (33% inhibition, p<0.001) of CD11c+ DCs carrying FITC were found in Hpse-KO mice.

### MMP-14 is Overexpressed and MMP-9 is Downregulated in DCs from *Hpse*-KO Mice

**Figure 6:**
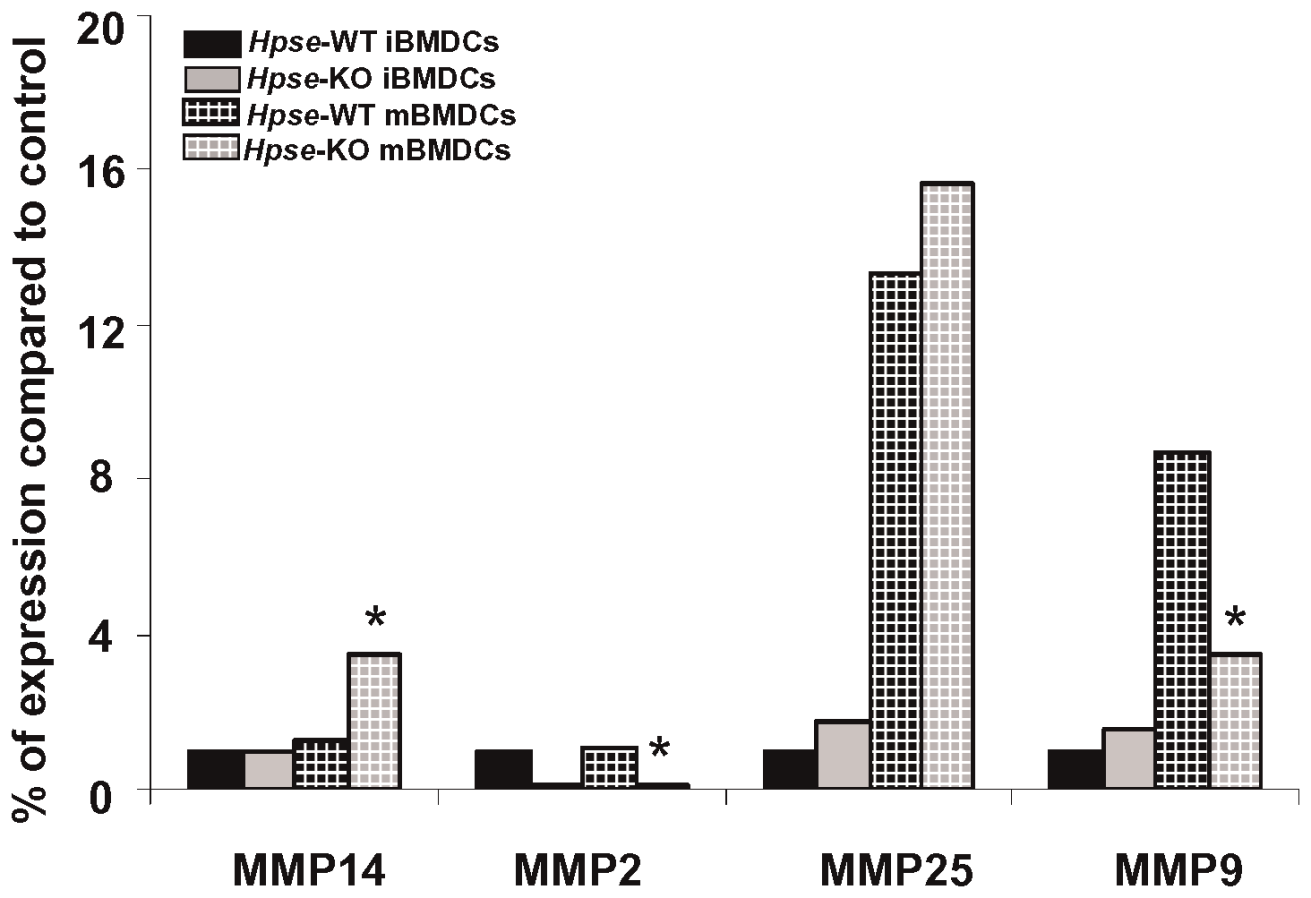
MMP expression. RT-PCR of MMPs in iBMDCs (black), and mBMDCs (grilled black), from *Hpse-*WT or in iBMDCs (gray), mBMDCs (grilled gray), from *Hpse*-KO mice. Maturation of BMDC was achieved with a cocktail of 30 ng/ml recombinant mTNFα and 1 µg/ml PGE2 for 24 hrs. RNA was extracted from iBMDCs and mBMDCs from *Hpse-*WT and *Hpse*-KO mice. Primers specific for MMP-2, MMP-9, MMP-25, and MMP-14 were used for RT-PCR (see Methods). Asterisk indicates p<0.02, t-test.

The unexpected result of only reduced migration, despite the fact that heparanase is unique in its ability to degrade heparan sulfate, led us to investigate whether other ECM degrading enzyme(s) were compensating for the lack of heparanase expression. Taking into account that matrix metalloproteinases (MMPs) play important role in rearranging the ECM structure, and thereby in tissue remodeling, morphogenesis, and neovascularization, we investigated the expression of MMPs by real-time PCR. For this purpose, total RNA extracted from BMDC of *Hpse-*KO and WT mice was analyzed using specific primers corresponding to MMP-2, -9,-25, and -14. As shown in Figure 6, MMP-14 was overexpressed in mBMDCs from *Hpse*-KO mice whereas MMP-9 was decreased in mBMDCs from *Hpse*-KO mice.

## Discussion

DCs are highly mobile cells with a superior capacity to recognize and take up antigens to initiate an immune response. It has been suggested that, in their immature stage of development, DCs patrol in the periphery as sentinels to sample foreign- or self-antigens, and, following specific pattern recognition receptors, undergo phenotypical and functional changes to differentiate into either their tolerogenic or stimulatory mature stage. At this point DCs readily migrate to the lymph nodes to present processed antigens to T cells [23], [24]. Trafficking of both immature and mature DCs is crucial for the execution of their functions, and depends mainly on chemotactic attraction and ECM degradation-mediated migration. Chemotaxis is critically modulated by chemokine responsiveness, mainly based on CCR7 and its two ligands, CCL19 and CCL21 [25]. ECM degradation in DC trafficking is poorly understood and was suggested to be achieved primarily by heparanase [16], and possibly MMP-14 [26] or other undermined metallproteinases.

In this study, as anticipated from our previous research [16], transmigration capacity in mBMDCs from *Hpse*-KO mice was significantly reduced both in vitro and in vivo, most probably due to the fact that ECM degradation is an important part of DC transmigration. However, despite the unique role of heparanase, transmigration was only reduced and not completely abolished. This is most probably explained by the compensatory higher expression of MMP-14 and CCR7 seen in BMDCs from *Hpse*-KO mice. MMPs constitute a family of proteinases that participate in cell migration [27]. MMPs may be expressed on the surface of cells, thus allowing for precise, localized proteolysis that creates a path for migrating cells [28]. Migration of both Langerhans cells and dermal DCs was prevented by broad spectrum MMP inhibitors (BB-3103), and by the natural tissue inhibitors of metalloproteinases (TIMP), TIMP-1 and TIMP-2 [29]. In addition, transmigration was rather delayed than inhibited as seen by the numbers of DCs reaching the lymph node 48 hours following the injection. At 48 hours following the injection it was anticipated that the DC number will decline [30], [31], as was seen in the wild type. However, BMDCs from *Hpse*-KO mice were found in higher numbers 48 hours following injection indicating delayed arrival.

Different cells express different MMPs as compensation for heparanase deficiency. Whereas DCs showed upregulation of MMP-14 and downregulation of MMP-2 and MMP-9, other tissues showed 2–3.5 fold overexpression of MMP-2 in many samples extracted from *Hpse-*KO mice, but not in DCs. For example, MMP-14 was overexpressed by 4–7 fold in the liver and kidney, but was downregulated approximately 4 fold in mammary glands derived from *Hpse-*KO mice. MMP-9 and MMP-25 expression levels were altered as well, depending on the tissue [17]. MMP-9, reported to be important in DC migration [32], [33], was surprisingly downregulated in contrast to MMP-14 upregulation, indicating perhaps different roles in transmigration. Upregulation of MMP-14 is quite unique and specific, at least in DCs. As a membrane-anchored protease on the cell surface, MMP-14 is ideally positioned for efficient proteolysis of a wide variety of substrates in the pericellular space. MMP-14 has been shown to possess strong proteolytic activity, and is capable of degrading ECM molecules, however, its role in modulating immune cell migration is still poorly understood. So far it has been shown that MMP-14 can directly modulate the migration of human monocytes [34], and it has been suggested play a role in DC degradation of the ECM [26].

Interestingly, cells transfected with active heparanase exhibited a marked decrease in MMP-14 expression, as a mirror image of the increased expression found in *Hpse-*KO mice. In contrast, transfecting MDA-231 cells with mutant inactive heparanase did not affect MMP expression [17], indicating that heparanase enzymatic activity is involved in the observed regulation of MMP expression. Taken together, this data may suggest that heparanase and MMP-14 play synergistic and compensatory roles in DC trafficking and may compensate for one another.

Another player is probably CCR7, which is a major molecule, important for chemotaxis-based DC homing and migration to the lymph nodes; in its absence, DCs exhibit impaired migration [2], [3], [25], [31], [35]. Although it is not an ECM enzyme, CCR7 may have, in a manner similar to MMP-14, a close relationship with heparanase, as a molecule that compensates for the lack of a heparan sulfate-degrading enzyme. These relationships between a chemotactic migratory- and a transmigratory molecule are novel and thus far unknown, and could indicate a close relationship between two related functions.

DCs from *Hpse-*KO mice exhibited dissociation between an increased mature phenotype and reduced transmigration capacity. The phenotype represented true maturation, with capability of pro-inflammatory cytokine secretion. It contrasted with the deficiency in E-cadherin, where DCs exhibited the so-called semi-mature phenotype, with increased MHC-class II and costimulatory molecules, but without capability of pro-inflammatory cytokine secretion [21].

It is not clear why costimulatory and IA/IE molecules are upregulated in DCs from these mice, even in their immature state. Is it the absence of heparanase, which may play an unknown nuclear regulatory role [16], or could it be related to CCR7 upregulation? Indeed, CCL19, a CCR7 ligand, has been shown to induce DC maturation, resulting in upregulation of costimulatory molecules and the production of proinflammatory cytokines [36]. Interestingly, despite the mature phenotype, apoptotic cell uptake was increased rather than decreased in DCs from *Hpse-KO* mice.

In summary, in vitro and in vivo transmigration is significantly delayed in DCs from *Hpse*-KO mice, but not completely abolished, most probably due to the observed upregulation of MMP-14 and CCR7. Dissociation between increased maturation phenotype and impaired transmigration is observed and may be related, at least in part, to CCR7 expression. Heparanase is an important enzyme for DC transmigration, and controls DC trafficking, together with CCR7 and its ligands, probably MMP-14 and possibly additional MMPs.

## Materials and Methods

### Reagents

Na_2_[^35^S]O_4_ was purchased from GE Healthcare Bioscience (Uppsala, Sweden). The bone marrow-derived DC medium consisted of RPMI 1640 (Invitrogen-Gibco, Carlsbad, CA) supplemented with 1% L-glutamine (Biological Industries, Kibbutz Beit Haemek, Israel), 1% penicillin/streptomycin (Biological Industries), 50 µM 2-mercaptoethanol (Sigma Aldrich, St Louis, MO) and 10% heat-inactivated FBS (Biological Industries). Recombinant murine granulocyte macrophage colony-stimulating factor (GMCSF) was obtained from Peprotech/Tebu (Frankfurt, Germany). Lipopolysaccharide (LPS), and mitomycin C, were obtained from Sigma Aldrich.

Recombinant mouse tumor necrosis factor-alpha (TNFα) was obtained from Prospect (Rehovot, Israel) and prostaglandin E2 (PGE2) from Cayman Chemical (Ann Arbor, MI). For the PCR reaction mix, qPCR SYBR Master Mix was purchased from Finnzymes (Espoo, Finland). For Western blot analysis, heparin-agarose beads, a mixture of protease inhibitors, FITC and FITC-labeled latex beads, and LPS were obtained from Sigma Aldrich, and chemiluminescent substrate WestOne is was purchased from iNtron Biotechnology (Gyeonggido, Korea). Matrigel for the Matrigel transmigration assay was provided by Collaborative Biomedical Products (Bedford, MA), recombinant mouse macrophage inflammatory protein-3 beta (mMIP-3β) was purchased from R&D Systems (Minneapolis, MN), and CytoCount beads were procured from Dako Cytomation (Glostrup, Denmark). 2.5 µM 5- and 6-carboxyfluorescein diacetate succinimidyl (CFSE) was obtained from Invitrogen –Gibco (Carlsbad, CA) Main fluorescein-conjugated Annexin V (Annexin V-FITC) and propidium iodide (PI) were obtained from MBL, Inc. (Woburn, MA). T cells were purified with the EasySep Kit, purchased from StemCell Technologies (Vancouver, Canada).

### Antibodies

Armenian hamster anti-mouse CD11c-PE, rat anti-mouse CCR7-PE, Armenian hamster anti-mouse CD80-FITC, rat anti-mouse CD86-FITC, Armenian hamster anti-mouse CD40-FITC, rat anti-mouse I-A/I-E-FITC, and Armenian hamster anti-mouse CD11c-FITC were purchased from BioLegend, Inc. (San Diego, CA). Isotype controls, Armenian hamster IgG-FITC, FITC Armenian hamster IgG-FITC, rat IgG2a, κ-FITC, Armenian hamster IgM-FITC, rat IgG2bκ -FITC, rat IgG2b, and κ-PE were purchased from BioLegend, Inc. Isotype control rat IgG2a-PE was obtained from Southern Biotech (Birmingham, AL). Goat anti-mouse actin was purchased from Sigma Aldrich.

For indirect immunofluorescence (frozen section), purified Armenian hamster anti-mouse CD11c, purified Armenian hamster IgG isotype, biotin Armenian hamster anti-mouse CD3ε and biotin Armenian hamster IgG isotype control were purchased from BioLegend, Inc. Goat anti-Armenian hamster Cy3 and streptavidin Cy5 were purchased from Jackson ImmunoResearch (West Grove, PA).

### Mice

Wild-type mice (*Hpse*-WT) and heparanase-deficient mice (*Hpse*-KO) were C57BL/6J background [17]. Mice were kindly provided by Professor Israel Vlodavsky (Hebrew University, Jerusalem) and the Bruce Rappaport Faculty of Medicine (Technion, Haifa, Israel). BALB/c mice (Harlan, Jerusalem, Israel) were used for T cell purification. All animal care protocols were approved by the animal committees of the Hebrew University, Jerusalem, Israel (MD-89.49-4) and the Biomedical Center, University of Uppsala, Sweden (C176/2), according to NIH guidelines [17].

1,1′-dioctadecyl-3,3,3′,3′-tetramethyl-indocarbocyanineperchlorate (DiI) was obtained from Molecular Probes.

### Splenic DC Subpopulation

Spleens were excised and placed in ice cold PBS. Tissues were mechanically minced with scissors. Cells were incubated for 3 min on ice and the supernatant was centrifuged at 1200 RPM, 4°C, 10 min to pellet. Hypotonic red blood cell lysis was performed following wash in PBS. Spleen cells were resuspended and stained with mouse-conjugated antibodies or isotype control mouse for 30 min on ice, and washed.

Antibodies for mouse spleen DC subpopulations: Armenian hamster anti-mouse CD11c-PE, rat anti-mouse CD45R/B220-APC, rat anti-mouse IA/IE-FITC, rat anti-mouse CD11b-APC/Cy7, and rat anti-mouse CD8α-PE/Cy7 were purchased from BioLegend, Inc. Isotype controls, Armenian hamster IgG-PE, rat IgG2a-APC, rat IgG2b-FITC, rat IgG2b-APC/Cy7, and rat IgG1-PE/Cy7 were purchased from BioLegend, Inc.

### Generation of Bone Marrow-derived DCs

BMDCs were generated according to the protocol of M.B. Lutz [37], with mild modifications. Briefly, the femurs and tibiae of female, 4–12 week old mice were removed and purified from surrounding muscle tissue. Thereafter, intact bones were left for disinfection in 70% ethanol for 1 min, washed twice in PBS, and transferred into a fresh dish with RPMI 1640. Then both ends of the bone were cut with scissors and the marrow was flushed with 2 ml of RPMI 1640 using a syringe and 25-gauge needle. Clusters within the marrow suspension were disintegrated by vigorous pipetting, and put on ice for 3 min to remove debris.

The cell culture medium was RPMI 1640-supplemented with 1% penicillin/streptomycin L-glutamine (2 mM), 2-mercaptoethanol (50 µM), and 10% heat-inactivated FBS. At day 0, BM leukocytes were seeded at 2×10^6^ cells per 100 mm dish in 10 ml cell culture medium containing 200 U/ml GMCSF. On day 3, another 10 ml cell culture medium containing 200 U/ml GMCSF was added to the plates. On days 6 and 8, half of the culture supernatant was collected and centrifuged, and the cell pellet was resuspended in 10 ml cell culture medium containing 200 U/ml GMCSF, and returned to the original plate. Maturation of BMDC was achieved with 0.5 µg/ml LPS or a cocktail of 30 ng/ml recombinant mTNFα and 1 µg/ml PGE2 for 24 hrs.

### Preparation of Dishes Coated with ECM

Cultures of bovine corneal endothelial cells were established from steer eyes and maintained in DMEM (1 g glucose/l) supplemented with 5% newborn calf serum, 10% FCS, and 1 ng/ml bFGF [38]. Bovine corneal endothelial cells were plated into 35 mm tissue culture dishes and cultured as described above, except that 4% dextran T-40 was included in the growth medium [7], [38]. Na_2_^35^SO_4_ (25 µCi/ml) was added on days 1 and 5 after seeding. On day 12, the subendothelial ECM was exposed by dissolving the cell layer with PBS containing 0.5% triton X-100 and 20 mM NH_4_OH, and washing four times with PBS. The ECM remained intact, free of cellular debris, and firmly attached to the entire area of the tissue culture dish [38]. Nearly 80% of ECM radioactivity was incorporated into heparan sulfate glycosaminoglycans.

### Heparanase Activity

To assess heparanase activity in whole cell lysates from Hpse-WT and from Hpse-KO mice, 6×10^6^ cells were lysed by three cycles of freezing and thawing, and incubated overnight at 37°C, pH 5.8, on ^35^S-labeled ECM, prepared as described above, with 150 µl heparanase reaction solution (0.15 M NaCl, 20 mM phosphate-citrate buffer, pH 5.8, 1 mM dithiothreitol [DTT], and 1 mM CaCl_2_). The incubation medium was centrifuged and the supernatant containing sulfate-labeled degradation fragments was analyzed by gel filtration on a 0.9×30 cm sepharose CL-6B column (Sigma Aldrich). Fractions (0.2 ml) were eluted with PBS, and their radioactivity was counted in a β-scintillation counter. Degradation fragments of heparan sulfate side chains were eluted from sepharose 6B at 0.5<K_av_<0.8 (fractions 15–30, peak II). Nearly intact heparan sulfate glycosaminoglycans were eluted at K_av_<0.2 (fractions 1–10, peak I) [7], [39], [40].

We used immobilized, sulfate-labeled ECM as a substrate, since it resembles the natural substrate degraded by the cells *in vivo* better than soluble heparin sulfate. Under this condition, decreased heparanase activity (i.e., a lower quantity of sulfate-labeled material eluted in peak II, fractions 15–30, 4–10 kDa) is not associated with an increase in the first peak (peak I), which corresponds to nondegraded heparan sulfate (M.W.>0.4×10^6^), kDa, or proteolytic activity, not related to heparanase. This is because the nondegraded, high molecular-weight material remains bound to the dish, and hence is not subjected to gel filtration analysis.

### RNA and RT-PCR Isolation

RNA was isolated with the RNeasy Mini Handbook (Quiagen GmbH, Hilden, Germany) according to the manufacturer’s instructions, and was quantified by ultraviolet absorption. After reverse transcription of 1 µg total RNA by oligo dT priming, the resulting single strand cDNA was amplified using TaqDNA polymerase (Promega, Madison, WI) and specific primers were directed against human heparanase (4U: 5′-ACA GTT CTA ATG CTC AGT TGC TC-3′ and 4L: 5′-AAA GAC GGC TAA GAT GCT GAA G-3′). PCR conditions were initial denaturation at 95°C for two minutes, denaturation at 96°C for 18 seconds, annealing for 80 seconds at 58°C, and extension for 70 seconds at 72°C (33 cycles). Aliquots (10 µl) of the amplified cDNA were separated by 1.5% agarose gel electrophoresis, visualized by ethidium bromide staining, and compared to the expression level of a ribosomal L-19 gene (L-19U: 5′- ATG CCA ACT CTC GTC AAC AG -3′; L-19 L: 5′- GCG CTT TCG TGC TTC CTT-3′ [40], [41]. Only RNA samples that gave completely negative results in PCR without reverse transcriptase were further analyzed.

### Flow Cytometry Analysis

Flow cytometry was performed on a FACScan™ flow cytometer (Becton Dickinson, Franklin Lakes, NJ), and data was analyzed using FCS Express V3 analysis software (DeNovo Software, Los Angeles, CA).

### *In vitro* Matrigel Transmigration Assay

Transwell inserts (6.5 mm) fitted with polycarbonate filters (5 µm pore, Corning Costar, High Wycombe, UK) were used. The upper surface of the filter was coated with 15 µg of matrigel, solubilized in 50 µl DDW, and air-dried for 1.5 hours in a hood. The lower compartment was filled with 600 µl of RPMI 1640 medium, supplemented in some experiments with 25–50 ng/ml recombinant mouse MIP-3β. BMDCs (50,000 cells/100 µl of RPMI) were then added to the upper compartment. The chambers were incubated at 37°C in 5% CO_2_ for 24 hours. Medium was collected from the lower compartment of the chamber and CytoCount beads were added in some experiments. An equal volume containing 20 µl of CytoCount beads, including transmigrated BMDCs, was added by reverse pipetting to the collected volume of the lower chambers in these experiments. CytoCount beads are easily distinguished from cells in the plot of side and forward scatter, and acquisition was stopped when 2,000 beads were counted. Results in these transmigration assays are presented as the number relative to 2,000 beads and not as a percent of input. Every experiment was performed in duplicate. For confirmation, counting beads were used to obtain cell counts. Since the beads had a high tendency to stick to the cells being studied, an indirect approach was pursued. Before and after every time point, a single tube with a known concentration of beads was vortexed for 5 seconds and then acquired 3 times. The quantity of beads corresponding to each sample tube was then calculated using a simple linear equation based on the average bead values. The quantity of cells gated was divided by the amount of beads in the sample.

Flow cytometry was performed on a FACScan™, and data was analyzed using FCS Express V3 analysis software (De Novo Software, Los Angeles, CA).

### *In vivo* Transmigration

Mature BMDCs (2×10^6^) were labeled with CFSE intracellular fluorescent dyes for 5 minutes at room temperature. After labeling, cells were washed extensively in PBS and injected subcutaneously into the footpads of *Hpse*-WT mice. Increased DC migration was elicited by subcutaneous pre-injection of 50 ng mTNFα into the footpad 24 hours before footpad injection of the labeled mBMDCs [31]. To analyze the number of migrating BMDCs, after 24 hours popliteal lymph nodes were extracted and minced into small fragments. Cells were analyzed for the presence of CD11c, PE, and CFSE+ by flow cytometry. CytoCount beads were used to evaluate cells that had migrated to the lymph nodes, as described previously. Flow cytometry was performed on a FACScan™, and data was analyzed using FCS Express V3 analysis software.

### FITC Skin Painting (FITC Transmigration)

FITC (Sigma Aldrich) was dissolved (5 mg/ml) in a 50∶50 (vol/vol) acetone-dibutylphthalate mixture just before application. 0.2 ml of the FITC solution was painted on the shaved abdomens of mice. Inguinal lymph nodes were obtained after 24 hours and mechanically disaggregated [22]. Cells were analyzed for the presence of CD11c-PE, and FITC^+^ by flow cytometry. Flow cytometry was performed on a FACScan™, and data was analysed using FCS Express V3 analysis software.

### Apoptotic Cell Interation with iDCs

Apoptotic cells were prepared as described elsewhere [42] and stained with Dii according to the manufacturer’s instructions. Labeled apoptotic cells were added to iDCs on day 6 of culture, for 1 or 2 h/37°C in 200 µl volume of culture media. In most assays, iDCs were labeled with CD11c-FITC. Uptake was read by a FACScan™. Uptake of FITC-latex beads (Sigma Aldrich), was used as a control for phagocytosis by CD11c.PE+ DCs.

### Cytokine Detection Assays

Cytokine production was assessed by analysis of culture supernatants of BMDCs from *Hpse*-KO and *Hpse*-WT mice after 24 h maturation (day 9) or 48 h maturation (day 10) with 10 ng/ml or 50 ng/ml or 100 ng/ml LPS, 10 µg/m or 5 µg/ml zymosan, or a cocktail of 30 ng/ml recombinant mTNFα and 1 µg/ml PGE2. Supernatant was collected and kept at −20°C for further analysis. ELISA kits from Diaclone Research (Besançon, France) were used for the detection of murine IL-6, murine IL-10, and murine TNFα. The ELISA Max Deluxe Set from BioLegend, Inc. was used for the detection of mouse IL-12/IL-23 p40. ELISA was performed according to the manufacturer’s instruction. Recombinant cytokines were used as standards, with curves generated from 15.6–500 pg/ml for murine IL-6, from 31.25–1000 pg/ml for murine IL-10, and from 7.8–500 pg/ml for TNFα, for mouse IL-12/IL-23 p40. Optical densities of the samples and standards were measured using the Sunrise ELISA Plate Reader (Tecan Group, Männedorf, Switzerland).

### Matrix Metalloproteinase Analysis

Real-time quantitative PCR analysis was performed with an automated rotor gene system RG-3000A (Corbett Research, Sydney, Australia). The PCR reaction mix (20 µl) was composed of 10 µl QPCR SYBR Master Mix and 5 µl of diluted cDNA (each sample in a six-plicate), with a final concentration of 0.3 µM of each primer. PCR conditions were as follows: an initial denaturation step at 95°C for 15 minutes; 40 cycles of denaturation at 94°C for 15 seconds, hybridization at 57°C for 30 seconds, and elongation at 72°C for 30 seconds. The primers MMP-2 (S: 5′- AGC GTG AAG TTT GGA AGC AT-3′ and AS: 5′- CAC ATC CTT CAC CTG GTG TG-3′), MMP-25 (S: 5′- GCT GAC TCG CTA TGG CTA CC-3′ and AS: 5′- GTC ATT GGG TCC ATT TGT CC-3′), MMP-14 (S: 5′- GCC TGG AAC ATT CTA ACG A-3′ and AS: 5′- CTT TGT GGG TGA CCC TGA CT-3′), and MMP-9 (S: 5′-AGA CGA CAT AGA CGG CAT CC-3′ and AS: 5′ GTG GTT CAG TTG TGG TGG TG-3′) were used. Actin primers (S: 5′- ATG CTC CCC GGG CTG TAT-3′ and AS: 5′- CAT AGG AGT CCT TCT GAC CCA TTC-3′) were used as an internal standard. The expression level of different MMPs in the *wt* tissue was regarded as 100%, and the MMP levels in the *Hpse-*KO mice were calculated relative to this value.

### Cell Staining, Tissue Histology, and Indirect Immunofluorescence

BMDCs were placed on slides by cytospin (2 min, 1000 rpm) and stained with Wright staining for light microscopy. Frozen sections of popliteal lymph nodes were fixed in 4% paraformaldehyde (10 min, room temperature) and stained with purified Armenian hamster anti-mouse CD11c or purified Armenian hamster IgG isotype control (1 h, room temperature), and detected with goat anti- Armenian hamster Cy3 (30 min, room temperature). Tissues were then stained with biotin Armenian hamster anti-mouse CD3 or biotin Armenian hamster IgG isotype control (1 hour, room temperature) and detected with streptavidin Cy5 (30 minutes, room temperature). Sections were viewed in confocal microscopy (zoom 60×5, Olympus IX70, Center Valley, PA).

### Statistical Analysis

SigmaPlot software, version 10.0 (Systat Software, Chicago, IL) was used to calculate statistical differences between groups, which were compared using the paired Student’s t-test). All p values were two-sided.
